# Timing, Dosage, and Adherence of Antiretroviral Therapy and Risk of Osteoporosis in Patients With Human Immunodeficiency Virus Infection in Taiwan: A Nested Case-Control Study

**DOI:** 10.3389/fphar.2021.631480

**Published:** 2021-04-30

**Authors:** Mu-Lin Chiu, Wen-Miin Liang, Ju-Pi Li, Chi-Fung Cheng, Jian-Shiun Chiou, Mao-Wang Ho, Yang-Chang Wu, Ting-Hsu Lin, Chiu-Chu Liao, Shao-Mei Huang, Fuu-Jen Tsai, Ying-Ju Lin

**Affiliations:** ^1^ Genetic Center, Department of Medical Research, China Medical University Hospital, Taichung, Taiwan; ^2^ School of Chinese Medicine, China Medical University, Taichung, Taiwan; ^3^ Department of Health Services Administration, China Medical University, Taichung, Taiwan; ^4^ School of Medicine, Chung Shan Medical University, Taichung, Taiwan; ^5^ Department of Pediatrics, Chung Shan Medical University Hospital, Taichung, Taiwan; ^6^ Section of Infectious Diseases, Department of Internal Medicine, China Medical University Hospital, Taichung, Taiwan; ^7^ Department of Biotechnology and Bioinformatics, Asia University, Taichung, Taiwan; ^8^ Graduate Institute of Integrated Medicine and Chinese Medicine Research and Development Center, China Medical University and Hospital, Taichung, Taiwan

**Keywords:** HIV, osteoporosis, antiretroviral therapy, usage timing, dosage, adherence

## Abstract

The progression of acquired immunodeficiency syndrome is delayed in patients with human immunodeficiency virus (HIV) infection receiving antiretroviral therapy (ART). However, long-term ART is associated with adverse effects. Osteoporosis is one of the adverse effects and is a multifactorial systemic skeletal disease associated with bone fragility and an increased risk of fracture. We performed a longitudinal, comprehensive, nested case-control study to explore the effect of ART on the risk of osteoporosis in 104 osteoporotic and 416 non-osteoporotic patients with HIV infection at their average age about 29 years old in Taiwan. Patients with history of ART, current exposure to ART, higher cumulative defined daily doses (DDDs), or higher ART adherence were at a higher risk of osteoporosis (*p* < 0.05). Patients receiving nucleoside/nucleotide reverse transcriptase inhibitor (NRTI)-containing regimen (zidovudine-lamivudine combination, lamivudine-abacavir combination, and abacavir alone) and protease inhibitor (PI)-containing regimen (lopinavir-ritonavir combination, ritonavir, and atazanavir) had a higher risk of osteoporosis (*p* < 0.05). Especially, patients receiving high doses of the PIs lopinavir-ritonavir combination had an increased risk of osteoporosis (*p* < 0.05). In conclusion, history of ART, current exposure to ART, higher cumulative DDDs, and higher ART adherence were associated with an increased risk of osteoporosis. Furthermore, NRTI- and PI-containing regimens and high doses of PIs lopinavir-ritonavir combination may be associated with an increased risk of osteoporosis in patients with HIV infection in Taiwan.

## Introduction

An estimated 37.9 million people were infected with human immunodeficiency virus (HIV)/acquired immunodeficiency syndrome (AIDS) as of 2018 (https://www.hiv.gov/hiv-basics/overview/data-and-trends/global-statistics). Among these, approximately 23.3 million were receiving antiretroviral therapy (ART). After ART, HIV replication is effectively inhibited and HIV-infected immune cells become latent (Pham and Mesplede, 2018; Zhang et al., 2019). Patients with HIV/AIDS who receive ART demonstrate delayed AIDS disease progression, improved quality of life, and lower all-cause mortality (Antiretroviral Therapy Cohort, 2017; Lu et al., 2018). These patients need to receive ART regularly on a lifelong basis.

Major ART drugs include nucleoside/nucleotide reverse transcriptase inhibitors (NRTIs) used alone or in combination with other antiviral drugs, such as protease inhibitors (PIs), non-nucleoside reverse transcriptase inhibitors (NNRTIs), or integrase strand transfer inhibitors (INSTIs). NRTIs include zidovudine, lamivudine, zidovudine-lamivudine combination, tenofovir disoproxil, lamivudine-abacavir combination, and abacavir; PIs include lopinavir-ritonavir combination, ritonavir, atazanavir, darunavir and tipranavir; NNRTIs include efavirenz, etravirine, rilpivirine, and nevirapine; INSTIs include dolutegravir and raltegravir. Long-term ART only suppresses virus replication and does not eliminate the virus. However, discontinuation of ART results in drug resistance, viral reactivation, and disease progression (Meintjes et al., 2017; Dubrocq and Rakhmanina, 2018). Long-term ART is associated with adverse effects such as hyperlipidemia, cardiovascular disease, osteoporosis, diabetes, and renal disease (Kwong et al., 2006; De Wit et al., 2008; Capeau et al., 2012; Achhra et al., 2016; Grant et al., 2016; Dorjee et al., 2017; Hoy et al., 2017; Tsai et al., 2017; Nan et al., 2018; Tsai et al., 2018).

Osteoporosis is a multifactorial systemic skeletal disease accompanied by low bone mineral density, deterioration of bone architecture, bone fragility, and consequently increased fracture risk (Ji and Yu, 2015; Sozen et al., 2017; Liu et al., 2019). Loss of bone mineral density is frequently observed in patients with HIV infection receiving ART (Duvivier et al., 2009; Van Vonderen et al., 2009; Grant et al., 2016; Hoy et al., 2017). Moreover, patients with HIV infection receiving PI-containing regimens demonstrate bone loss in the spine, whereas those receiving NRTI-containing regimens show bone loss in the hip (Hoy et al., 2017). Other studies have reported that a combination of NRTI drugs - and PI-containing regimens also lead to bone loss among patients with HIV infection (Duvivier et al., 2009; van Vonderen et al., 2009).

Previous studies have reported that patients with HIV infection receiving ART are at a higher risk of bone loss (Duvivier et al., 2009; Van Vonderen et al., 2009; Grant et al., 2016; Hoy et al., 2017). However, the correlation of ART usage, timing, dosages, and adherence with osteoporosis risk among patients with HIV infection remains unclear. In this longitudinal nested case-control study, a comprehensive database was used to explore the association between ART and the risk of osteoporosis among patients with HIV infection in Taiwan. Detailed associations between usage, timing, dosages, and adherence to ART and the risk of osteoporosis were investigated.

## Materials and Methods

### Study Cohort

**FIGURE 1:**
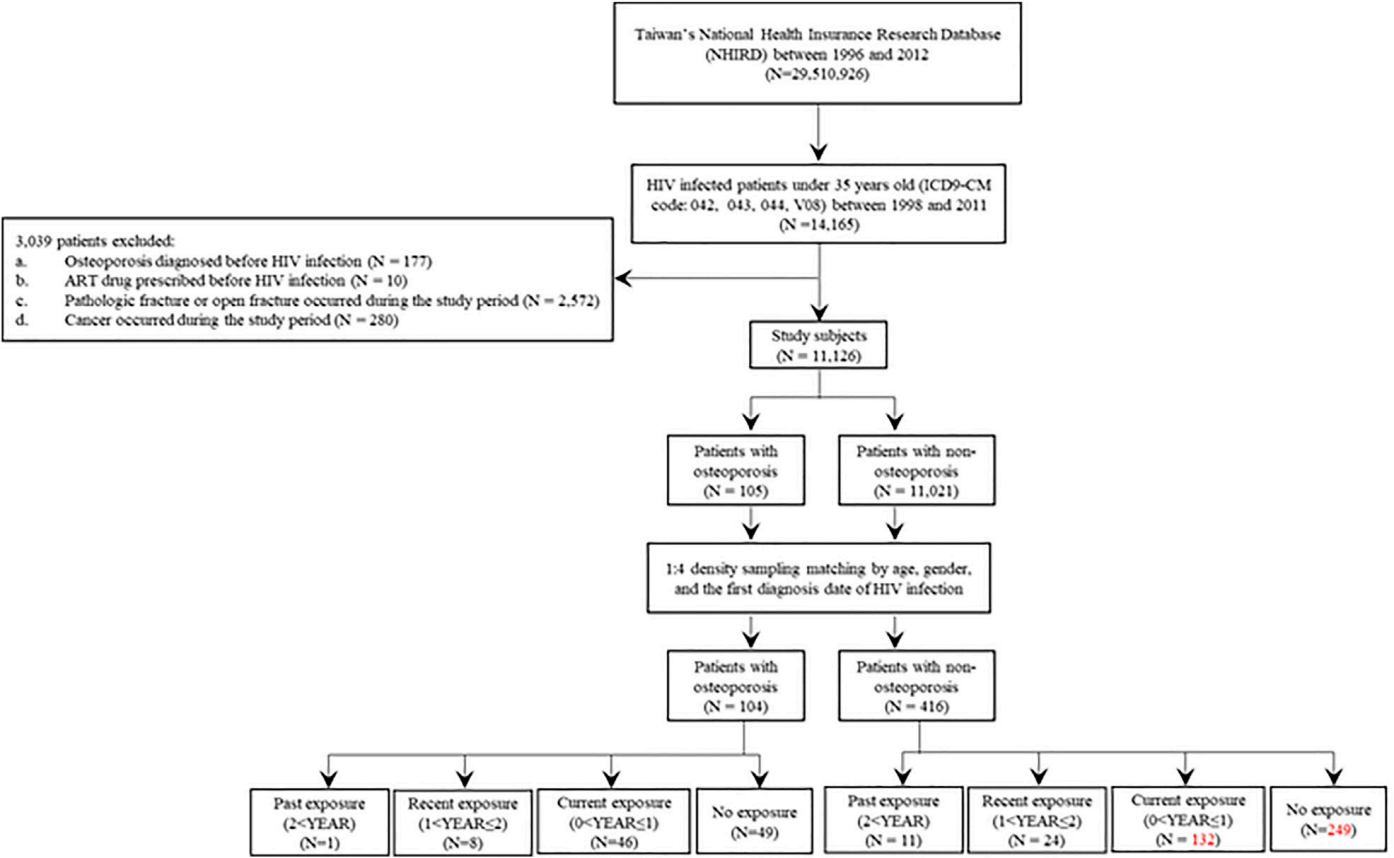
Flow chart for recruitment of osteoporosis cases and non-osteoporosis controls among patients with HIV infection. HIV, human immunodeficiency virus.

This nested case-control study was approved by the Human Studies Committee of China Medical University Hospital, Taichung, Taiwan (approval number: CMUH107-REC3-074). We identified 14,165 patients with HIV infection (International Classification of Diseases, Ninth Revision, Clinical Modification [ICD-9-CM]: codes 042, 043, 044, and V08) aged under 35 years whose data were recorded in the National Health Insurance Research database (http://nhird.nhri.org.tw/) between 1998 and 2011 (Figure 1).

A total of 11,126 patients were included in the study after applying the following exclusion criteria: patients with a diagnosis of osteoporosis before human immunodeficiency virus (HIV) infection (*N* = 177), patients with antiretroviral therapy (ART) prescriptions before HIV infection (*N* = 10), patients with a diagnosis of pathologic or open fracture during the study period (*N* = 2,572), or patients with cancers during the study period (*N* = 280).

Osteoporosis was defined according to the ICD-9-CM codes 7330, 7332-7337, and 7339. Pathologic or open fracture was defined according to the ICD-9-CM: codes 7331, 73381, 73382, 8055, 8071, 8089, 8101, 8111, 8123, 81351, 81391, 81392, 81393, 8141, 8151, 8161, 8209, 82031, 8211, 8221, 8239, 82391, 82392, 8249, 82539, 8261, 8281, 8479, 883, and 8832. Cancers were defined according to the ICD-9-CM: codes 140-172, 174-195, and 200-208.

### Cases and Controls

As cases, we included patients who had reported osteoporosis for the first time after HIV infection during the study period (*N* = 105) (Figure 1 and Table 1). The date of osteoporosis diagnosis was defined as the index date. The controls were patients with HIV infection who did not develop osteoporosis during the study period (*N* = 11,021) (Figure 1 and Table 1). To prevent potential bias, the density sampling matching method (1:4 ratio for cases and controls) was applied to match osteoporosis and non-osteoporosis groups based on age, sex, and the first date of HIV infection diagnosis. After matching, 104 osteoporosis cases and 416 non-osteoporosis controls were included in the study (Figure 1 and Table 1).

**TABLE 1:** Basic characteristics of HIV-infected patients in Taiwan.

	Total patients		*p*-value	Matched patients		*p*-value
			
	Osteoporosis (*N* = 105) *N* (%)	Non-osteoporosis (*N* = 11,021) *N* (%)		Osteoporosis (*N* = 104)*N* (%)	Non-osteoporosis (*N* = 416) *N* (%)	
Age (years old ± SD)	28.61± 5.76	27.07± 6.15	0.011	28.62± 5.78	28.82± 5.56	0.750
Sex	—	—	0.032	—	—	1.000
Male	72 (68.57%)	8527 (77.37%)	—	72 (69.23%)	288 (69.23%)	—
Female	33 (31.43%)	2494 (22.63%)	—	32 (30.77%)	128 (30.77%)	—
Follow-up years (Mean ± SD)	4.81± 3.12	6.29± 3.61	<0.001	4.78± 3.12	4.78± 3.11	1.000
Comorbidities	—	—	—	—	—	—
Myocardial infarction	0 (0.00%)	2 (0.02%)	0.890	0 (0.00%)	0 (0.00%)	NA
Congestive heart failure	0 (0.00%)	20 (0.18%)	0.662	0 (0.00%)	0 (0.00%)	NA
Peripheral vascular disease	1 (0.95%)	42 (0.38%)	0.348	1 (0.96%)	1 (0.24%)	0.288
Cerebrovascular disease	0 (0.00%)	42 (0.38%)	0.526	0 (0.00%)	1 (0.24%)	0.617
Dementia	0 (0.00%)	2 (0.02%)	0.890	0 (0.00%)	0 (0.00%)	NA
Chronic pulmonary disease	7 (6.67%)	474 (4.3%)	0.235	7 (6.73%)	10 (2.4%)	0.026
Rheumatic disease	4 (3.81%)	55 (0.5%)	<0.001	4 (3.85%)	2 (0.48%)	0.004
Peptic ulcer disease	6 (5.71%)	654 (5.93%)	0.924	6 (5.77%)	15 (3.61%)	0.316
Mild liver disease	8 (7.62%)	890 (8.08%)	0.864	8 (7.69%)	23 (5.53%)	0.405
Diabetes without chronic complications	4 (3.81%)	41 (0.37%)	<0.001	4 (3.85%)	0 (0.00%)	<0.001
Diabetes with chronic complication	2 (1.9%)	5 (0.05%)	<0.001	2 (1.92%)	0 (0.00%)	0.005
Hemiplegia or paraplegia	0 (0.00%)	16 (0.15%)	0.696	0 (0.00%)	1 (0.24%)	0.617
Renal disease	0 (0.00%)	20 (0.18%)	0.662	0 (0.00%)	1 (0.24%)	0.617
Moderate or severe liver disease	0 (0.00%)	26 (0.24%)	0.618	0 (0.00%)	0 (0.00%)	NA
ART usage	—	—	NA	—	—	0.027
No	NA	NA	—	48 (46.15%)	242 (58.17%)	—
Yes	NA	NA	—	56 (53.85%)	174 (41.83%)	—
NRTI-containing regimen	—	—	NA	—	—	0.127
No	NA	NA	—	75 (72.12%)	329 (79.09%)	—
Yes	NA	NA	—	29 (27.88%)	87 (20.91%)	—
PI-Containing regimen	—	—	NA	—	—	0.005
No	NA	NA	—	71 (68.27%)	337 (81.01%)	—
Yes	NA	NA	—	33 (31.73%)	79 (18.99%)	—
NNRTI-containing regimen	—	—	NA	—	—	0.922
No	NA	NA	—	75 (72.12%)	298 (71.63%)	—
Yes	NA	NA	—	29 (27.88%)	118 (28.37%)	—
Other ART-containing regimen	—	—	NA	—	—	0.288
No	NA	NA	—	103 (99.04%)	415 (99.76%)	—
Yes	NA	NA	—	1 (0.96%)	1 (0.24%)	—
Over two ART drugs-containing regimen	—	—	NA	—	—	0.015
No	NA	NA	—	52 (50%)	262 (62.98%)	—
Yes	NA	NA	—	52 (50%)	154 (37.02%)	—

N, number; ART, antiretroviral therapy; SD, standard deviation; HIV, human immunodeficiency virus; NA, not applicable.

Density sampling matching (1: 4 ratio) was performed using age, sex, and the first diagnosis date of HIV infection.

*p* Values were obtained by the chi-square test; *p* values for age and the follow-up years were obtained by the un-paired Student’s t test.

Significant *p-*values (*p* < 0.05) are indicated in bold and italic font.

Comorbidities present before the date of HIV infection diagnosis were defined as follows: Myocardial infarction (ICD-9-CM:410.x, 412*),congestive heart failure (ICD-9-CM: 428.x), peripheral vascular disease (ICD-9-CM: 441.x* , 443.9* , 785.4* , V43.4* , 38.48(P)), cerebrovascular disease (ICD-9-CM: 430.x-438.x*), dementia (ICD-9-CM: 290.x*), chronic pulmonary disease (ICD-9-CM: 490.x-496.x* , 500.x-505.x* , 506.4*), rheumatic disease (ICD-9-CM: 710.0, 710.1*, 710.4*, 714.0–714.2*, 714.81, 725.x*), peptic ulcer disease (ICD-9-CM: 531.x-534.x), mild liver disease (ICD-9-CM: 571.2*, 571.4–571.6*), diabetes without chronic complication (ICD-9-CM: 250.0–250.3*, 250.7*), diabetes with chronic complication (ICD-9-CM: 250.4–250.6*), hemiplegia or paraplegia (ICD-9-CM: 344.1, 342.x), renal disease (ICD-9-CM: 582.x*, 583–583.7*, 585.x*, 586.x*, 588.x*), and moderate or severe liver disease (ICD-9-CM: 456.0–456.21*, 572.2–572.8*).

### Exposure to ART Drugs

**FIGURE 2:**
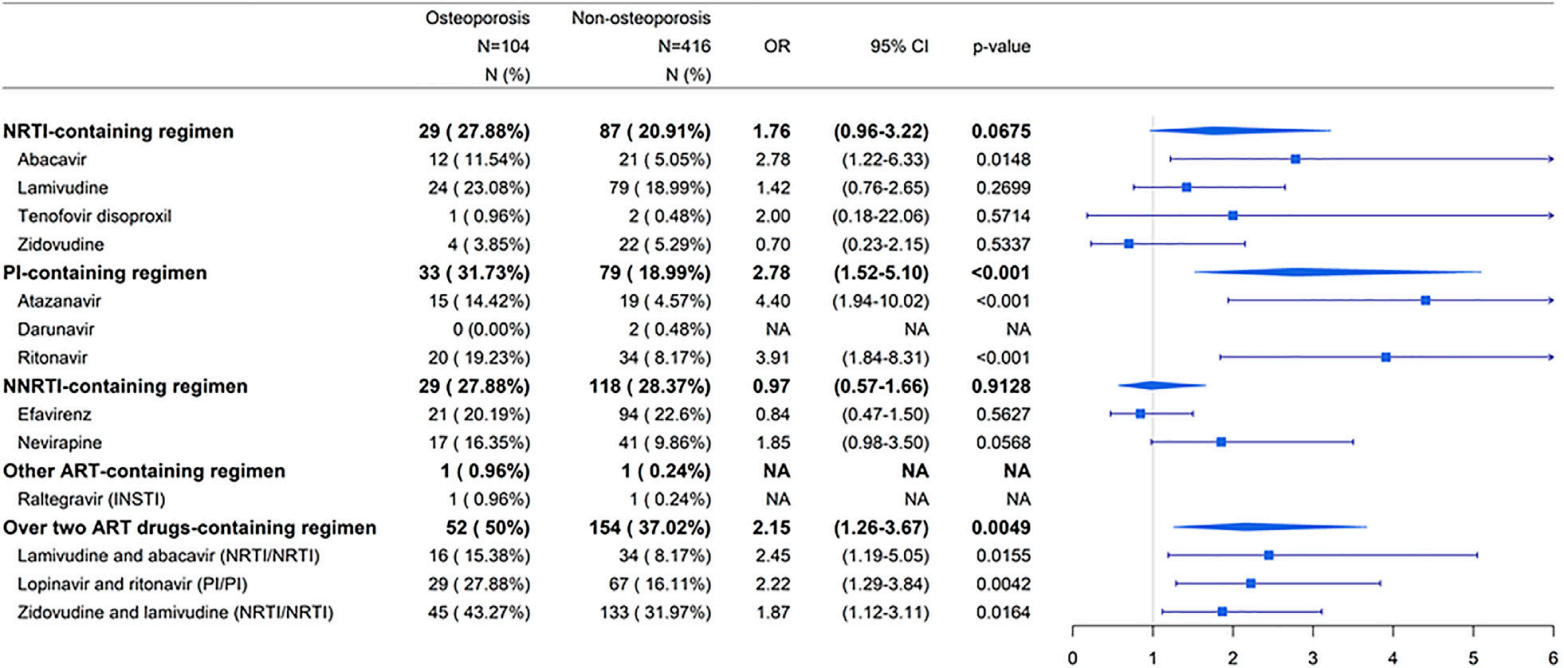
Summarized associations between individual ART drugs and risk of osteoporosis compared to non-ART use. Abbreviations: ART, antiretroviral therapy; NRTI, nucleoside/nucleotide reverse transcriptase inhibitor; PI, protease inhibitor; NNRTI, non-nucleoside reverse transcriptase inhibitor; INSTI, integrase strand transfer inhibitor; N, number; OR, odds ratio; CI, confidence interval; NA, not applicable.

Exposure was defined as the usage of ART drugs during the study period (Table 2). The ART drugs prescribed for patients with HIV infection were as follows: nucleoside/nucleotide reverse transcriptase inhibitor (NRTI) ATC code: J05AF06, J05AF02, J05AF05, J05AF04, J05AF07, J05AF01, J05AF03; protease inhibitor (PI) ATC code: J05AE08, J05AE10, J05AE02, J05AE04, J05AE03, J05AE01, and J05AE09; non-nucleoside reverse-transcriptase inhibitor (NNRTI) ATC code: J05AG03, J05AG04, J05AG01, and J05AG05; integrase strand transfer inhibitor (INSTI) ATC code: J05AX12, J05AX07, J05AX09, and J05AX08. For the ATC code of over two ART drugs in one tablet: J05AR02, J05AR10, J05AR01, J05AR03, J05AR14, J05AR21, J05AR20, J05AR19, J05AR22, J05AR18, J05AR06, J05AR08, J05AR25, J05AR13, J05AR04, and J05AR05 (Supplementary Table S1). The currently used ART drugs and also available in our database were shown (Figure 2; Supplementary Table S2).

**TABLE 2:** Risk of osteoporosis when exposure to ART drugs.

			OR	95% CI	*p-value*
	Osteoporosis *N* = 104	Non-osteoporosis *N* = 416			
	*N* (%)	*N* (%)			
ART drugs	—	—	—	—	—
Non-exposure	48 (46.15%)	242 (58.17%)	Ref	Ref	Ref
Exposure	56 (53.85%)	174 (41.83%)	2.11	(1.22–3.66)	0.0077
Timing of exposure	—	—	—	—	—
Non-exposure	49 (47.12%)	249 (59.86%)	Ref	Ref	Ref
Current exposure (0 < YEAR ≤1)	46 (44.23%)	132 (31.73%)	2.51	(1.38–4.56)	0.0026
Recent exposure (1 < YEAR ≤2)	8 (7.69%)	24 (5.77%)	2.42	(0.94–6.26)	0.0674
Past exposure (2 < YEAR)	1 (0.96%)	11 (2.64%)	0.65	(0.08–5.33)	0.6855
Cumulative defined daily dose (DDD)	—	—	—	—	—
Non-exposure	49 (47.12%)	249 (59.86%)	Ref	Ref	Ref
Low (cumulative DDDs < 2500)	24 (23.08%)	72 (17.31%)	2.26	(1.14–4.50)	0.0194
High (cumulative DDDs ≥ 2500)	31 (29.81%)	95 (22.84%)	2.48	(1.22–5.04)	0.0117
Adherence	—	—	—	—	—
Non-exposure	49 (47.12%)	249 (59.86%)	Ref	Ref	Ref
Low (0 < ADH ≤ 0.8)	32 (30.77%)	100 (24.04%)	2.32	(1.22–4.42)	0.0107
High (0.8 < ADH)	23 (22.12%)	67 (16.11%)	2.43	(1.21–4.89)	0.0125

N, number; ART, antiretroviral therapy; OR, odds ratio; CI, confidence interval; Ref, reference; DDD, defined daily dose; ADH, adherence.

Conditional logistic regression model was performed with adjustments for age and comorbidities.

Significant *p*-values (*p<0.05*) are indicated in bold and italic font.

Cumulative defined daily dose (DDD) and adherence were calculated during the period between the date of HIV infection diagnosis and the date of osteoporosis diagnosis.

The definition of cumulative DDD: The total dose of drug given to a patient from the start of drug usage to the study end.

The definition of adherence: (Total number of prescribed days of a patient from the start of drug usage to the study end)/(total number of days of observation of a patient from the start of drug to the study end).

**FIGURE 3:**
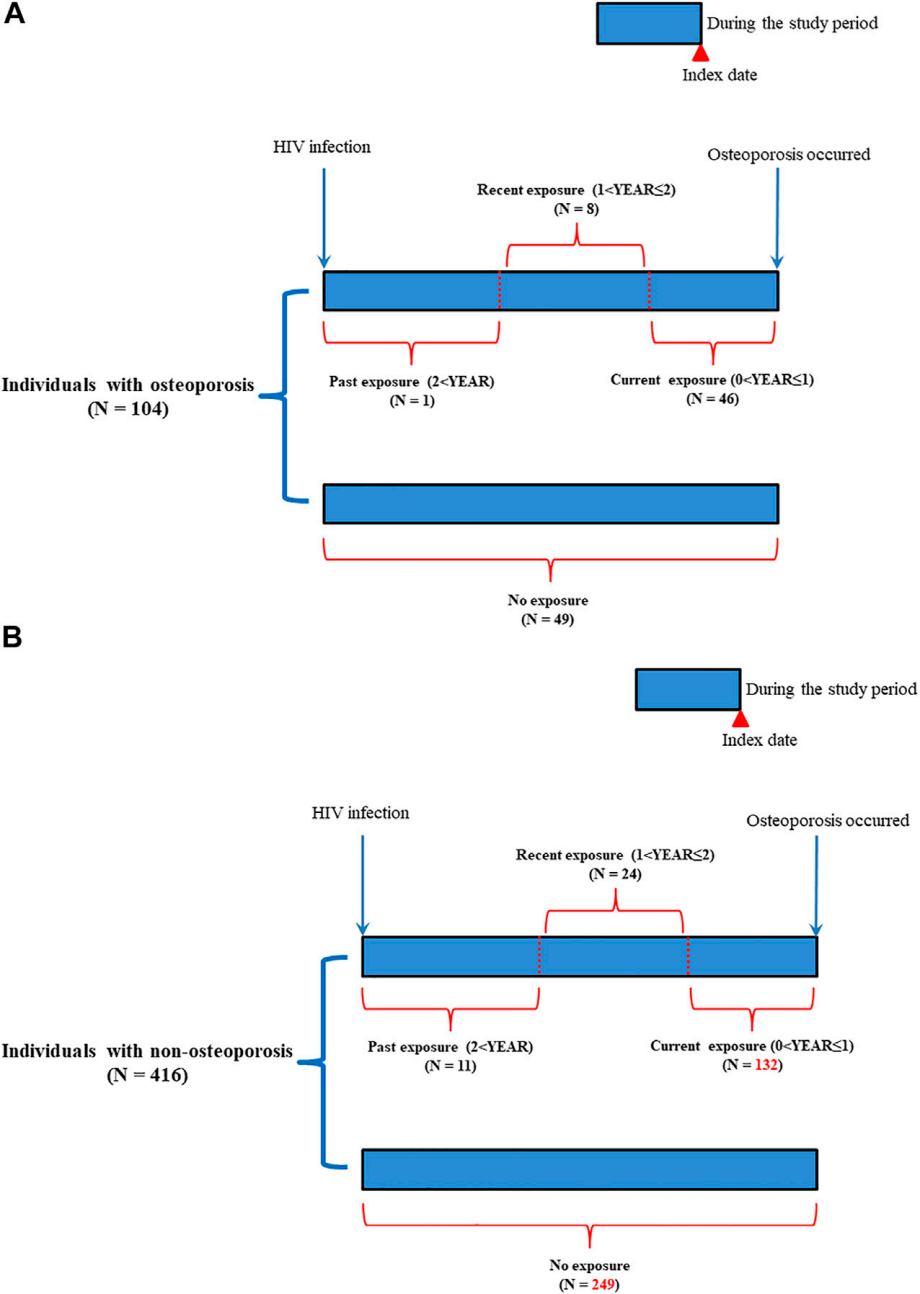
Follow-up time for patients with HIV infection. **(A)**: Patients with osteoporosis; **(B)**: Patients with non-osteoporosis.

For determining the duration of exposure to ART drugs, we further categorized exposure into non-exposure, current exposure (0 < YEAR ≤ 1), recent exposure (1 < YEAR ≤ 2), and past exposure (2 < YEAR) groups based on the “latest” ART prescription date prior to the index date (Table 2 and Figure 3). The index date was defined as the diagnosed date of osteoporosis. For the current exposure patients, patients were defined if they had ART drug availability during the time window of 0-1 year before the index date (Figure 3). For the recent exposure patients, patients were defined if they had ART drug availability during the time window of 1-2 years before the index date (Figure 3). For the past exposure patients, patients were defined if they had ART drug availability during the time window of more than 2 years before the index date (Figure 3). Current exposure patients included the patients who continuously used ART drugs since HIV infection (Figure 3). For the cumulative defined daily dose (DDD), we further categorized cumulative DDD (usage dose) into non-use, cumulative DDDs < 2500, and cumulative DDDs ≥ 2500 during the study period. For adherence to ART, we further classified the usage adherence into non-exposure, low adherence (0 < adherence (ADH) ≤0.8), and high adherence (0.8 < ADH) during the study period.

### Patient Data Collection

The data (the National Health Insurance Research database) included longitudinal inpatient and outpatient records of age, sex, admission, prescription, diagnosis, procedures, and ambulatory care. In this study, demographic data (age, sex, follow-up period, comorbidities, and ART usage) were collected for patients with HIV infection (Table 1). Comorbidities were present before the date of HIV infection diagnosis. Comorbidities included myocardial infarction (ICD-9-CM:410. x, 412*), congestive heart failure (ICD-9-CM: 428. x), peripheral vascular disease (ICD-9-CM: 441. x*, 443.9*, 785.4*, V43.4*, 38.48(P)), cerebrovascular disease (ICD-9-CM: 430. x-438. x*), dementia (ICD-9-CM: 290. x*), chronic pulmonary disease (ICD-9-CM: 490. x-496. x*, 500. x-505. x*, 506.4*), rheumatic disease (ICD-9-CM: 710.0, 710.1*, 710.4*, 714.0-714.2*, 714.81, 725. x*), peptic ulcer disease (ICD-9-CM: 531. x-534. x), mild liver disease (ICD-9-CM: 571.2*, 571.4-571.6*), diabetes without chronic complications (ICD-9-CM: 250.0-250.3*, 250.7*), diabetes with chronic complications (ICD-9-CM: 250.4-250.6*), hemiplegia or paraplegia (ICD-9-CM: 344.1, 342. x), renal disease (ICD-9-CM: 582. x*, 583-583.7*, 585. x*, 586. x*, 588. x*), and moderate or severe liver disease (ICD-9-CM: 456.0-456.21*, 572.2-572.8*).

Cumulative DDD and adherence were calculated during the study period. Cumulative DDD was defined as the total dose of ART administered to a patient from the initiation of ART treatment to the end of study. Adherence was defined as follows: (total number of prescribed days from ART initiation to study end)/(total number of observation days for a patient from ART initiation to study end).

### Statistical and Subgroup Analyses

**FIGURE 4:**
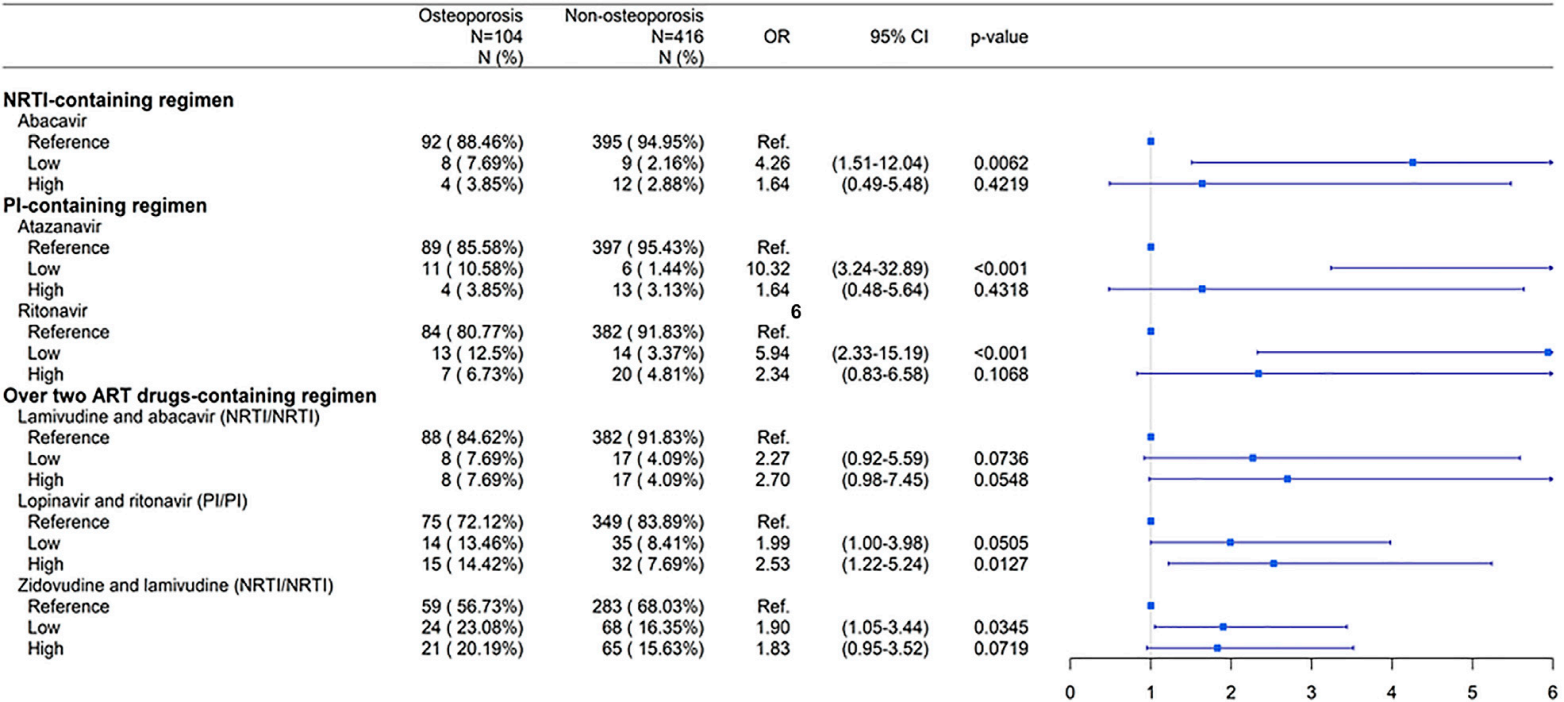
Dose-response relationship between individual ART drugs and risk of osteoporosis compared to non-ART use. Low: low cumulative defined daily doses (DDDs) < 2500; High: high cumulative DDDs ≥ 2500. Abbreviations: ART, antiretroviral therapy; NRTI, nucleoside/nucleotide reverse transcriptase inhibitor; PI, protease inhibitor; N, number; OR, odds ratio; CI, confidence interval.

Categorical variables, including sex, comorbidities, and ART usage, were analyzed using the Chi-square test between osteoporosis cases and non-osteoporosis controls (Table 1). Continuous variables, including age and follow-up period, were analyzed using Student’s *t-*test (Table 1). Conditional logistic regression analysis was applied to investigate the association between ART use and osteoporosis risk in patients with HIV infection (Table 2; Figure 2; Figure 4). Subgroup analyses according to NRTI-, PI-, NNRTI-, other ART-containing, and over two ART drugs-containing regimens were used to investigate the association between ART use and osteoporosis risk during the study period (Figure 2). Subgroup analyses for specific NRTI- and PI- containing regimens with dose-response relationships were performed to explore the association between the dose effect of specific NRTI- and PI- containing regimens and osteoporosis risk (Figure 4). Odds ratios (ORs) and 95% confidence intervals (CIs) were calculated. Results with *p*-values < 0.05 were considered significant. Statistical analyses were performed using the Statistical Analysis System software (version 9.3; SAS Institute, Cary, NC).

## Results

### Basic Characteristics of Study Subjects

The study subjects comprised 105 patients with osteoporosis and 11,021 non-osteoporosis controls (Figure 1; Table 1). Significant differences were observed regarding age, sex, follow-up period, and comorbidities between the two groups (*p* < 0.05). Patients with osteoporosis included more women, were older, had shorter follow-up years, and had increased incidence of comorbidities (rheumatic disease and diabetes with or without chronic complications). Furthermore, the matched osteoporosis cases included a higher number of cases of chronic pulmonary disease, rheumatic disease, and diabetes with or without chronic complications (Table 1). The exposure to antiretroviral therapy (ART) was defined as the use of ART drugs during the study period (Figures 1, 3).

### ART Usage, Timing, Dosage, and Adherence and Osteoporosis Risk in Patients With HIV Infection

Conditional logistic regression analysis was used to investigate the association between ART usage and osteoporosis risk adjusted for age and comorbidities (Table 2).

As shown in Table 2, there were significant differences in ART usage, duration, dosage, and adherence (*p* < 0.05). The patients who received ART had a higher risk of osteoporosis, with an odds ratio (OR) of 2.11 (95% confidence interval (CI): 1.22-3.66) than those who did not use ART during the study period (*p* = 0.0077; Table 2). Patients with current exposure to ART had a higher risk of osteoporosis, with an OR of 2.51 (95% CI: 1.38-4.56) than those without exposure to ART (*p* = 0.0026) (Table 2). However, the associations were not statistically significant among patients with recent or past exposures (*p* > 0.05) (Table 2).

Patients receiving cumulative defined daily doses (DDDs) ≥ 2500 had higher risk of osteoporosis, with an OR of 2.48 (95% CI: 1.22-5.04), than those not receiving ART (*p* = 0.0117) (Table 2). Patients receiving cumulative DDDs < 2500 also had a risk of osteoporosis, but it was lower (OR, 2.26; 95% CI: 1.14-4.50) than in those not receiving ART (*p* = 0.0194) (Table 2). Patients with ART adherence > 0.8 had higher risk of osteoporosis, with an OR of 2.43 (95% CI: 1.21-4.89), than those not receiving ART (*p* = 0.0125; Table 2). Although patients with 0 < ART adherence ≤ 0.8 had a risk of osteoporosis, it was lower (OR, 2.32; 95% CI: 1.22-4.42) than in patients not receiving ART (*p* = 0.0107) (Table 2).

These results suggest that patients were at higher risk of osteoporosis when they received ART, were currently exposed, had higher cumulative DDDs, or had higher ART adherence.

### NRTI-, PI-, and NNRTI-Containing Regimens and Risk of Osteoporosis in Patients With HIV Infection

To investigate which types of ART-containing regimens were associated with increased risk of osteoporosis, patients receiving nucleoside/nucleotide reverse transcriptase inhibitor (NRTI)-, protease inhibitor (PI)-, non-nucleoside reverse transcriptase inhibitor (NNRTI)-, other ART-containing, and over two ART drugs-containing regimens were investigated (Supplementary Table S1). The currently used ART drugs and available in our database were shown (Figure 2). For the NRTI-containing regimen, abacavir were associated with higher risks of osteoporosis in patients receiving ART than in those not receiving ART (*p* < 0.05) (Figure 2). Patients receiving PI-based ART had a higher risk of osteoporosis, with an OR of 2.78 (95% CI: 1.52-5.10), than those not receiving ART (*p* < 0.001) (Figure 2). Among the PI drugs, ritonavir and atazanavir were associated with higher risks of osteoporosis in patients receiving ART than in those not receiving ART (*p* < 0.001) (Figure 2). There were no sufficient numbers in darunavir (PI)- and raltegravir (integrase strand transfer inhibitor (INSTI))-containing regimens. The association was not statistically significant among patients receiving NNRTIs (*p* > 0.05) (Figure 2). For over two ART drugs-containing regimens, lamivudine and abacavir (NRTI/NRTI), lopinavir and ritonavir (PI/PI), and zidovudine and lamivudine (NRTI/NRTI) were associated with higher risks of osteoporosis in patients receiving ART than in those not receiving ART (*p* < 0.05) (Figure 2).

These results suggest that patients were at an increased risk of osteoporosis when they received NRTI- and PI-containing regimens. Patients receiving the NRTIs zidovudine-lamivudine combination, lamivudine-abacavir combination, or abacavir were at a risk of osteoporosis. Patients receiving the PIs lopinavir-ritonavir combination, ritonavir, and atazanavir were at an elevated risk of osteoporosis.

### Dose-Response Relation of NRTI-Containing and PI-Containing Regimens and Risk of Osteoporosis in Patients With HIV Infection

Three NRTIs, zidovudine-lamivudine combination, lamivudine-abacavir combination, and abacavir, were associated with an increased risk of osteoporosis (*p* < 0.05) (Figure 2). However, patients receiving higher doses of these drugs had no significantly increased risk of osteoporosis compared with those not receiving ART (Figure 4).

Three PIs lopinavir-ritonavir combination, ritonavir, and atazanavir were associated with an increased risk of osteoporosis (*p* < 0.05) (Figure 2). Patients receiving higher doses of lopinavir-ritonavir combination were at a significantly higher risk of osteoporosis than those not receiving ART (Figure 4).

These results suggest that patients were at an increased risk of osteoporosis when they had higher doses of the PI-containing regimen lopinavir-ritonavir. However, patients with higher doses of NRTIs had no significantly increased risk of osteoporosis.

## Discussion

In this nested case-control study, we investigated the association between antiretroviral therapy (ART) and the risk of osteoporosis in patients with human immunodeficiency virus (HIV) infection in Taiwan. We found that ART usage, current exposure, higher cumulative defined daily doses (DDDs), and higher ART adherence were associated with an increased risk of osteoporosis. Furthermore, nucleoside/nucleotide reverse transcriptase inhibitor (NRTI)- and protease inhibitor (PI)-containing regimens and higher doses of PIs–lopinavir-ritonavir combination—may be associated with an increased risk of osteoporosis in patients with HIV infection in Taiwan. Therefore, this study showed that ART, especially NRTI-containing and PI-containing regimens, may be potential risk factors for osteoporosis. Switching from ART regimens may be one option to improve bone health in patients with HIV infection (Ciccullo et al., 2018).

In our study, patients with HIV infection were at a higher risk of osteoporosis at a relatively young age. ART usage, current exposure, higher cumulative DDDs, and higher ART adherence were associated with osteoporosis risk. Osteoporosis, characterized by low bone mineral density and deterioration of bone architecture, is usually associated with old age and is more prevalent in women (Kanis et al., 2008). The current exposure patients were those who had ART drug availability during the time window of 0-1 year before osteoporosis. Current exposure patients included the patients who continuously used ART drugs since HIV infection. Why current exposure to ART were associated with higher risk of osteoporosis may be due to patients may have higher cumulative DDDs since HIV infection. Bone mineral density screening has been suggested for male patients with HIV infection older than 50 years and in postmenopausal women (Aberg et al., 2014; Gonciulea et al., 2017). In agreement with our results, previous study findings reveal that bone loss is observed in patients with HIV infection who receive ART (Mccomsey et al., 2008; Duvivier et al., 2009; Van Vonderen et al., 2009; Haskelberg et al., 2012; Grant et al., 2016; Hoy et al., 2017; Komatsu et al., 2018).

Our results demonstrated that patients receiving NRTI-containing regimen had a higher risk of osteoporosis. Studies have reported that NRTI-containing regimens, including zidovudine-lamivudine combination therapy and tenofovir monotherapies, induce osteoporosis or osteoporotic fractures (Van Vonderen et al., 2009; Haskelberg et al., 2012; Grant et al., 2016; Komatsu et al., 2018). Furthermore, our study showed that patients receiving zidovudine-lamivudine combination, lamivudine-abacavir combination, and abacavir had a higher risk of osteoporosis. In agreement with our results, van Vonderen et al. reported that patients with HIV infection treated with zidovudine and lamivudine combination had greater bone loss (Van Vonderen et al., 2009). Zidovudine and lamivudine stimulate osteoclastogenesis *in vitro* and cause osteopenia in mice by increasing the promoter activity of tartrate-resistant acid phosphatase and the binding of nuclear factor-kappa B (Pan et al., 2004; Pan et al., 2006).

Our results showed that patients receiving PI-containing regimen had a higher risk of osteoporosis. Among the PIs, lopinavir-ritonavir combination, ritonavir, and atazanavir were associated with a higher risk of osteoporosis. Furthermore, our results suggested that high doses of three PIs lopinavir-ritonavir combination were associated with an increased risk of osteoporosis. In agreement with our results, previous findings revealed lower bone mineral density in patients treated with ritonavir (Dimeglio et al., 2013). Increased osteoclast activity has been observed in the presence of ritonavir (Jain and Lenhard, 2002). Reportedly, ritonavir enhances osteoclast differentiation via upregulation of the production of osteoclast growth factors (Modarresi et al., 2009).

The limitations of this study include not considering factors such as genetic background information (both virus genotype and human genetics); environmental factors (nutrition level, education, job stress, and exercise); and other clinical characteristics, including body mass index, bone density, blood HIV viral load, blood cluster of differentiation antigen 4 (CD4) count, bone mineral density test results (for example: dual energy x ray absorptiometry (DXA)-based examinations), and serum bone turnover markers. Another limitations of this study include the lack of information on current therapies, such as integrase inhibitors or tenofovir alafenamide (TAF)-based therapies (Supplementary Table S1).

In conclusion, this was a longitudinal and broad study that elucidated the association of ART with the risk of osteoporosis using a database of patients with HIV infection in Taiwan. Patients receiving NRTI- or PI-containing regimens have a higher risk of osteoporosis. These patients are suggested to have regular examinations for bone mineral density when they are administrated with NRTI- or PI-containing regimens. This information may help identify therapeutic options regarding long-term ART for the prevention of adverse effects, particularly osteoporosis, in patients with HIV infection.

## Data Availability

The original contributions presented in the study are included in the article/Supplementary Material, further inquiries can be directed to the corresponding authors.
