# Supplementary material for: Timing, Dosage, and Adherence of Antiretroviral Therapy and Risk of Osteoporosis in Patients With Human Immunodeficiency Virus Infection in Taiwan: A Nested Case-Control Study
Source: Front Pharmacol. 2021 Apr 30;12:631480. doi: 10.3389/fphar.2021.631480 (PMC8121495; doi:10.3389/fphar.2021.631480)
Supplement: Supplementary file 1 [file DataSheet1.docx]

**Supplementary Information**

Supplementary Text

Tables S1-S2

**Supplementary Text**

**TABLE S1 | Risk of osteoporosis when exposure to ART-containing regimens in the database**.

**TABLE S2 | Frequency distribution of ART drug usage among patients with HIV infection**.

| **TABLE S1 \| Risk of osteoporosis when exposure to ART-containing regimens in the database** | | | | | | | | | | | |
| --- | --- | --- | --- | --- | --- | --- | --- | --- | --- | --- | --- |
|  | **ATC code** | **Available in Taiwan (1996-2012)** | **Currently available in Taiwan (2021)** | **Available in the database in Taiwan (1996-2012)** | **Available in the database in Taiwan (1996-2012)** | | | | | | |
|  |  |  |  |  | **Osteoporosis N = 104** |  | **Non-osteoporosis N = 416** |  | **OR** | **95% CI** | ***p-value*** |
|  |  |  |  |  | **N (%)** |  | **N (%)** |  |  |  |  |
| **NRTI-containing regimen** |  |  |  |  | 29 ( 27.88%) |  | 87 ( 20.91%) |  | 1.76 | (0.96-3.22) | 0.068 |
| Abacavir | J05AF06 | 1 | 1 | 1 | 12 ( 11.54%) |  | 21 ( 5.05%) |  | 2.78 | (1.22-6.33) | ***0.015*** |
| Didanosine | J05AF02 | 1 | 0 | 1 | 13 ( 12.5%) |  | 38 ( 9.13%) |  | 1.51 | (0.72-3.13) | 0.274 |
| Lamivudine | J05AF05 | 1 | 1 | 1 | 24 ( 23.08%) |  | 79 ( 18.99%) |  | 1.42 | (0.76-2.65) | 0.270 |
| Stavudine | J05AF04 | 1 | 0 | 1 | 20 ( 19.23%) |  | 53 ( 12.74%) |  | 1.97 | (0.99-3.89) | 0.052 |
| Tenofovir disoproxil | J05AF07 | 1 | 1 | 1 | 1 ( 0.96%) |  | 2 ( 0.48%) |  | 2.00 | (0.18-22.06) | 0.571 |
| Zalcitabine | J05AF03 | 1 | 0 | 1 | 2 ( 1.92%) |  | 4 ( 0.96%) |  | 2.17 | (0.35-13.47) | 0.404 |
| Zidovudine | J05AF01 | 1 | 1 | 1 | 4 ( 3.85%) |  | 22 ( 5.29%) |  | 0.70 | (0.23-2.15) | 0.534 |
| **PI-containing regimen** |  |  |  |  | 33 ( 31.73%) |  | 79 ( 18.99%) |  | 2.78 | (1.52-5.1) | ***<0.001*** |
| Atazanavir | J05AE08 | 1 | 1 | 1 | 15 ( 14.42%) |  | 19 ( 4.57%) |  | 4.40 | (1.94-10.02) | ***<0.001*** |
| Darunavir | J05AE10 | 1 | 1 | 1 | 0 (0.00%) |  | 2 ( 0.48%) |  | NA | NA | NA |
| Indinavir | J05AE02 | 1 | 0 | 1 | 12 ( 11.54%) |  | 36 ( 8.65%) |  | 1.52 | (0.68-3.39) | 0.302 |
| Nelfinavir | J05AE04 | 1 | 0 | 1 | 8 ( 7.69%) |  | 11 ( 2.64%) |  | 3.18 | (1.21-8.33) | ***0.019*** |
| Ritonavir | J05AE03 | 1 | 1 | 1 | 20 ( 19.23%) |  | 34 ( 8.17%) |  | 3.91 | (1.84-8.31) | ***<0.001*** |
| Saquinavir | J05AE01 | 1 | 0 | 1 | 15 ( 14.42%) |  | 25 ( 6.01%) |  | 5.22 | (1.91-14.24) | ***0.001*** |
| Tipranavir | J05AE09 | 1 | 1 | 0 | NA |  | NA |  | NA | NA | NA |
| **NNRTI-containing regimen** |  |  |  |  | 29 ( 27.88%) |  | 118 ( 28.37%) |  | 0.97 | (0.57-1.66) | 0.913 |
| Efavirenz | J05AG03 | 1 | 1 | 1 | 21 ( 20.19%) |  | 94 ( 22.6%) |  | 0.84 | (0.47-1.5) | 0.563 |
| Etravirine | J05AG04 | 1 | 1 | 0 | NA |  | NA |  | NA | NA | NA |
| Nevirapine | J05AG01 | 1 | 1 | 1 | 17 ( 16.35%) |  | 41 ( 9.86%) |  | 1.85 | (0.98-3.5) | 0.057 |
| Rilpivirine | J05AG05 | 0 | 1 | 0 | NA |  | NA |  | NA | NA | NA |
| **Other ART-containing regimen** |  |  |  |  | 1 ( 0.96%) |  | 1 ( 0.24%) |  | NA | NA | NA |
| Dolutegravir (INSTI) | J05AX12 | 0 | 1 | 0 | NA |  | NA |  | NA | NA | NA |
| Enfuvirtide (Fusion inhibitor) | J05AX07 | 1 | 0 | 0 | NA |  | NA |  | NA | NA | NA |
| Maraviroc (CCR5 receptor inhibitor) | J05AX09 | 1 | 1 | 0 | NA |  | NA |  | NA | NA | NA |
| Raltegravir (INSTI) | J05AX08 | 1 | 1 | 1 | 1 ( 0.96%) |  | 1 ( 0.24%) |  | NA | NA | NA |
| **Over two ART drugs-containing regimen** |  |  |  |  | 52 ( 50%) |  | 154 ( 37.02%) |  | 2.15 | (1.26-3.67) | ***0.005*** |
| Lamivudine and abacavir (NRTI/NRTI) | J05AR02 | 1 | 1 | 1 | 16 ( 15.38%) |  | 34 ( 8.17%) |  | 2.45 | (1.19-5.05) | ***0.016*** |
| Lopinavir and ritonavir (PI/PI) | J05AR10 | 1 | 1 | 1 | 29 ( 27.88%) |  | 67 ( 16.11%) |  | 2.22 | (1.29-3.84) | ***0.004*** |
| Zidovudine and lamivudine (NRTI/NRTI) | J05AR01 | 1 | 1 | 1 | 45 ( 43.27%) |  | 133 ( 31.97%) |  | 1.87 | (1.12-3.11) | ***0.016*** |
| Darunavir and cobicistat (PI/PI) | J05AR14 | 0 | 1 | 0 | NA |  | NA |  | NA | NA | NA |
| Dolutegravir and rilpivirine (INSTI/NNRTI) | J05AR21 | 0 | 1 | 0 | NA |  | NA |  | NA | NA | NA |
| Emtricitabine, tenofovir alafenamide and bictegravir (NRTI/NRTI/INSTI) | J05AR20 | 0 | 1 | 0 | NA |  | NA |  | NA | NA | NA |
| Emtricitabine, tenofovir alafenamide and rilpivirine (NRTI/NRTI/NNRTI) | J05AR19 | 0 | 1 | 0 | NA |  | NA |  | NA | NA | NA |
| Emtricitabine, tenofovir alafenamide, darunavir and cobicistat (NRTI/NRTI/PI/PI) | J05AR22 | 0 | 1 | 0 | NA |  | NA |  | NA | NA | NA |
| Emtricitabine, tenofovir alafenamide, elvitegravir and cobicistat (NRTI/NRTI/INSTI/PI) | J05AR18 | 0 | 1 | 0 | NA |  | NA |  | NA | NA | NA |
| Emtricitabine, tenofovir disoproxil and efavirenz (NRTI/NRTI/NNRTI) | J05AR06 | 0 | 1 | 0 | NA |  | NA |  | NA | NA | NA |
| Emtricitabine, tenofovir disoproxil and rilpivirine (NRTI/NRTI/NNRTI) | J05AR08 | 0 | 1 | 0 | NA |  | NA |  | NA | NA | NA |
| Lamivudine and dolutegravir (NRTI/INSTI) | J05AR25 | 0 | 1 | 0 | NA |  | NA |  | NA | NA | NA |
| Lamivudine, abacavir and dolutegravir (NRTI/NRTI/INSTI) | J05AR13 | 0 | 1 | 0 | NA |  | NA |  | NA | NA | NA |
| Lamivudine, tenofovir disoproxil and doravirine (NRTI/NRTI/NNRTI) | J05AR24 | 0 | 1 | 0 | NA |  | NA |  | NA | NA | NA |
| Tenofovir disoproxil and emtricitabine (NRTI/NRTI) | J05AR03 | 0 | 1 | 0 | NA |  | NA |  | NA | NA | NA |
| Zidovudine, lamivudine and abacavir (NRTI/NRTI/NRTI) | J05AR04 | 1 | 0 | 0 | NA |  | NA |  | NA | NA | NA |
| Zidovudine, lamivudine and nevirapine (NRTI/NRTI/NNRTI) | J05AR05 | 1 | 1 | 0 | NA |  | NA |  | NA | NA | NA |
| N, number; ART, antiretroviral therapy; NRTI, nucleoside/nucleotide reverse transcriptase inhibitor; PI, protease inhibitor; NNRTI, non-nucleoside reverse-transcriptase inhibitor; INSTI, integrase strand transfer inhibitor; OR, odds ratio; CI, confidence interval; NA, not applicable. | | | | | | | | | | | |
| Conditional logistic regression model was performed with adjustments for age and comorbidities. | | | | | | | | | | | |
| Significant *p*-values (*p<0.05*) are indicated in bold and italic font. | | | | | | | | | | | |
| The usage of NRTI-containing, PI-containing, NNRTI-containing, other ART-containing, and over two ART drugs-containing regimens was counted as the HIV-infected patients who were prescribed during the study period in the database. | | | | | | | | | | | |
| The study period was defined between the date of HIV infection diagnosis and the date of osteoporosis diagnosis. | | | | | | | | | | | |
| The database used in this study was between 1996 and 2012. | | | | | | | | | | | |

| **TABLE S2** \| Frequency distribution of ART drug usage among patients with HIV infection | | | |
| --- | --- | --- | --- |
| **ART drugs** | **ATC code** | **Frequency of prescriptions (during the study period)** | |
|  |  | **N** | **%** |
| **NRTI-containing regimen** |  | **7304** | **28.04** |
| Abacavir | J05AF06 | 867 | 3.33 |
| Lamivudine | J05AF05 | 2914 | 11.19 |
| Tenofovir disoproxil | J05AF07 | 37 | 0.14 |
| Zidovudine | J05AF01 | 121 | 0.46 |
| **PI-containing regimen** |  | **3523** | **13.53** |
| Atazanavir | J05AE08 | 769 | 2.95 |
| Darunavir | J05AE10 | 63 | 0.24 |
| Ritonavir | J05AE03 | 963 | 3.7 |
| **NNRTI-containing regimen** |  | **5478** | **21.03** |
| Efavirenz | J05AG03 | 3924 | 15.07 |
| Nevirapine | J05AG01 | 1554 | 5.97 |
| **Other ART-containing regimen** |  | **27** | **0.1** |
| Raltegravir (INSTI) | J05AX08 | 27 | 0.1 |
| **Over two ART drugs-containing regimen** |  | **9714** | **37.3** |
| Lamivudine and abacavir (NRTI/NRTI) | J05AR02 | 991 | 3.8 |
| Lopinavir and ritonavir (PI/PI) | J05AR10 | 2697 | 10.35 |
| Zidovudine and lamivudine (NRTI/NRTI) | J05AR01 | 6026 | 23.14 |
| N, number; ART, antiretroviral therapy; HIV, human immunodeficiency virus; NRTI, nucleoside/nucleotide reverse transcriptase inhibitor; PI, protease inhibitor; NNRTI, non-nucleoside reverse-transcriptase inhibitor; INSTI, integrase strand transfer inhibitor. | | | |
| The exposure to ART drugs may be occurred before any time of the index date. The usage of NRTI-containing, PI-containing, NNRTI-containing, other ART-containing, and over two ART drugs-containing regimens was counted as the HIV-infected patients who were prescribed during the study period in the database. The study period was defined between the date of HIV infection diagnosis and the date of osteoporosis diagnosis. | | | |
| The total prescription frequencies = 26,046. | | | |
| The database used in this study was between 1996 and 2012. | | | |
